# Acacetin inhibits inflammation by blocking MAPK/NF-κB pathways and NLRP3 inflammasome activation

**DOI:** 10.3389/fphar.2024.1286546

**Published:** 2024-02-08

**Authors:** Juan Bu, Yeledan Mahan, Shengnan Zhang, Xuanxia Wu, Xiaoling Zhang, Ling Zhou, Yanmin Zhang

**Affiliations:** ^1^ Medical and Translational Research Center, People’s Hospital of Xinjiang Uygur Autonomous Region, Urumqi, China; ^2^ Scientific Research and Education Center, People’s Hospital of Xinjiang Uygur Autonomous Region, Urumqi, China

**Keywords:** inflammasome, NLRP3, acacetin, MAPK pathway, NF-κB pathway

## Abstract

**Objective:** Our preliminary research indicates that acacetin modulates the nucleotide-binding oligomerization domain (NOD)-like receptor pyrin domain containing 3 (NLRP3) inflammasome, providing protection against Alzheimer’s Disease (AD) and cerebral ischemic reperfusion injury. The mechanisms of acacetin to inhibit the activation of the NLRP3 inflammasome remain fully elucidated. This study aims to investigate the effects and potential mechanisms of acacetin on various agonists induced NLRP3 inflammasome activation.

**Methods:** A model for the NLRP3 inflammasome activation was established in mouse bone marrow-derived macrophages (BMDMs) using Monosodium Urate (MSU), Nigericin, Adenosine Triphosphate (ATP), and Pam3CSK4, separately. Western blot analysis (WB) was employed to detect Pro-caspase-1, Pro-Interleukin-1β (Pro-IL-1β) in cell lysates, and caspase-1, IL-1β in supernatants. Enzyme-Linked Immunosorbent Assay (ELISA) was used to measured the release of IL-1β, IL-18, and Tumor Necrosis Factor-alpha (TNF-α) in cell supernatants to assess the impact of acacetin on NLRP3 inflammasome activation. The lactate dehydrogenase (LDH) release was also assessed. The Nuclear Factor Kappa B (NF-κB) and Mitogen-Activated Protein Kinase (MAPK) signaling pathways related proteins were evaluated by WB, and NF-κB nuclear translocation was observed via laser scanning confocal microscopy (LSCM). Disuccinimidyl Suberate (DSS) cross-linking was employed to detect oligomerization of Apoptosis-associated Speck-like protein containing a Caspase Recruitment Domain (ASC), and LSCM was also used to observe Reactive Oxygen Species (ROS) production. Inductively Coupled Plasma (ICP) and N-(6-methoxyquinolyl) acetoethyl ester (MQAE) assays were utilized to determined the effects of acacetin on the efflux of potassium (K+) and chloride (Cl-) ions.

**Results:** Acacetin inhibited NLRP3 inflammasome activation induced by various agonists, reducing the release of TNF-α, IL-1β, IL-18, and LDH. It suppressed the expression of Lipopolysaccharides (LPS)-activated Phosphorylated ERK (p-ERK), p-JNK, and p-p38, inhibited NF-κB p65 phosphorylation and nuclear translocation. Acacetin also reduced ROS production and inhibited ASC aggregation, thus suppressing NLRP3 inflammasome activation. Notably, acacetin did not affect K+ and Cl-ions efflux during the activation process.

**Conclusion:** Acacetin shows inhibitory effects on both the priming and assembly processes of the NLRP3 inflammasome, positioning it as a promising new candidate for the treatment of NLRP3 inflammasome-related diseases.

## 1 Introduction

Inflammation represents a biological paradox. While moderate inflammatory responses can confer benefits to the host, aiding in the combat against foreign pathogen invasions, excessive or chronic inflammation may lead to tissue damage and consequential pathological changes. The nucleotide-binding oligomerization domain (NOD)-like receptor pyrin domain containing 3 (NLRP3) inflammasome plays a critical role in these inflammatory responses. Comprised of Apoptosis-associated Speck-like protein containing a Caspase Recruitment Domain (ASC), NLRP3, and pro-caspase-1, the NLRP3 inflammasome, upon activation, catalyzes the activation of caspase-1, which mediates the discharge of inflammatory cytokines Interleukin-1β (IL-1β) and Interleukin-18 (IL-18), thereby leading to inflammation and pyroptosis (Xu Y. et al., 2023; Ma, 2023). Studies support the implication of mitogen-activated protein kinase (MAPK) and Nuclear Factor Kappa-B (NF-κB) pathways in modulating NLRP3 inflammasome activation, thereby participating in the inflammatory response (He et al., 2013; Robblee et al., 2016). The activation of NLRP3 inflammasome correlates with a variety of diseases, including peritonitis (Qin and Zhao, 2023), arthritis (Wang et al., 2023), Alzheimer’s disease (AD) (Lv et al., 2023), and ischemic stroke (Han and Le, 2023), etc. Hence, the therapeutic efficacy of NLRP3 inflammasome inhibitors, which have manifested significant prognostic improvements in animal models of these diseases, underscores the imperative for their discovery.

At present, identified inhibitors of NLRP3 inflammasome encompass agents such as sulforaphane (Kiser et al., 2021), oridonin (He et al., 2018), BAY11-7082 (Irrera et al., 2017), INF39 (Shi et al., 2021), MCC950 (Luo et al., 2019), CY-09 (Wang et al., 2022), INF-200 (Gastaldi et al., 2023), SB-222200 (Zhou et al., 2023), Alantolactone (Li W. et al., 2023), and Tabersonine (Xu H.-W. et al., 2023), among others. Nevertheless, these therapeutic agents encounter drawbacks including limited specificity, short half-lives, suboptimal bioavailability, and an uncertain clinical application outlook. Acacetin, also referred to as robinin, is a flavonoid compound extracted from various plants, including chrysanthemums, *Robinia pseudoacacia*, and *Saussurea involucrata*. This compound, characterized by its small molecular weight, ability to penetrate the blood-brain barrier, minimal toxicity, and extensive pharmacological activities (Singh et al., 2020). Our previous study (Bu et al., 2019) revealed that acacetin mitigated ischemic stroke in mouse models by inhibiting microglial overactivation, modulating the NF-κB/NLRP3 pathway, and downregulating inflammatory cytokines such as TNF-α, IL-1β, and IL-6. In addition, acacetin showed significant neuroprotective properties by diminishing infarct volume and ameliorating neurological scores in these models. *In vitro* study, acacetin increased the survival rate of microglial cells following the oxygen-glucose deprivation and reoxygenation (OGD/R) injury, decreased lactate dehydrogenase (LDH) release, stimulated autophagy, and inhibited NLRP3 inflammasome activation (Bu et al., 2023). Furthermore, acacetin has demonstrated an ability to impede NLRP3 inflammasome activation, reduce inflammatory cytokine release, attenuate senile plaques development in AD mice, improve cognitive and exploratory abilities in AD mice, and thereby impart a protective role against AD (Bu et al., 2022). Given the critical role that NLRP3 inflammasome plays in the onset and progression of inflammatory diseases and the significant protective effects of acacetin on these diseases, questions arise regarding the potential relationship between acacetin’s protective role and its capacity to inhibit NLRP3 inflammasome activation.

Herein, we initially isolated macrophages from mouse bone marrow to explore the impact of acacetin on the activation of NLRP3 inflammasome induced by both canonical (i.e., by activators monosodium urate (MSU), Nigericin, and adenosine triphosphate (ATP)) and non-canonical pathways. The aim was to clarify whether acacetin could suppress NLRP3 inflammasome activation, thus exerting anti-inflammatory effects. Subsequently, we investigated whether acacetin impacts the NF-κB and MAPK pathways, sequentially inhibiting the priming of NLRP3 inflammasome. Following this, the study employed Disuccinimidyl Suberate (DSS) cross-linking to detect ASC oligomerization and laser scanning confocal microscopy (LSCM) to monitor the production of Reactive Oxygen Species (ROS). The impact of acacetin on potassium (K+) and chloride (Cl-) ions efflux was quantified using Inductively Coupled Plasma (ICP) and N-(6-methoxyquinolyl) acetoethyl ester (MQAE) assays, respectively. These methods facilitated a precise understanding of acacetin’s role in the assembly and upstream processes of the NLRP3 inflammasome.

## 2 Materials and methods

### 2.1 Cell preparation, stimulation, and grouping

Bone marrow-derived macrophages (BMDMs) were isolated from 6–8 weeks old C57BL/6J mice supplied by Xinjiang Medical University (License Number: SCXK (Xin) 2018–0002). Following euthanization and sterilization, both femurs and tibias were excised and the bone marrow was flushed into DMEM (C11965500BT, Gibco, United States) medium using sterile phosphate buffer saline (PBS, ZLI-9062, ZSGB-Bio, China). The obtained cell suspension was centrifuged at 110 *g* for 10 min at room temperature, after which the supernatant was discarded. The resultant cell pellet was resuspended in 2 mL of red blood cell lysis buffer and maintained on ice for 5 min. Subsequently, 8 mL of DMEM supplemented with 10% fetal bovine serum (FBS, FND500, Excell Bio, China) and 1% penicillin-streptomycin (15070–063, Gibco, United States) was added. After mixing, the solution was centrifuged at 220 *g* (with a centrifuge radius of 10 cm) for 5 min at room temperature. The supernatant was discarded, and the cells were then resuspended in DMEM containing 10 ng/mL of Granulocyte-Macrophage Colony Stimulating Factor (GM-CSF, P00184, Solarbio, Beijing, China), 10% FBS, and 1% penicillin-streptomycin. Ensuring a homogenous suspension through repeated pipetting, the cells were cultured in a 37°C incubator. After 3 days in culture, a complete medium replacement was conducted, followed by a half medium change on day 5. Microscopic examination revealed that the adherent cells had satisfactory attachment, extending pseudopodia, and adopted a spindle shape.

When cell confluency reached 80%, BMDMs cells (5 × 10^5^) were seeded to a 12-well plate and then treated with LPS (50 ng/mL, L2630, Sigma, United States) for 3 h. Afterwards, the cells were treated with Acacetin (00017, Sigma, United States) for 0.5 h and respectively stimulated with MSU (150 μg/mL, tlrl-msu/MSU-41–07, InvivoGen, France) for 4 h, Nigericin (10 μM, tlrl-nig, InvivoGen, France) for 0.5 h, and ATP (2.5 mM, A2383-5G/SLCD1216, Sigma, United States) for 0.5 h. For the activation of non-canonical pathways, cells were pretreated with Pam3CSK4 (400 ng/mL, Tlrl-pms/PMS-41-03, InvivoGen, France) for 3 h, then treated with Acacetin for 0.5 h, and then transfected with LPS (500 ng/mL) using Lipofectamine 3000 (L3000-008/2218558, ThermoFisher, United States of America) for 16 h. Both the supernatant and cell pellets were collected for subsequent analyses.

Groups for assessing acacetin’s impact on NF-κB and MAPK signaling pathways included: a control group, groups treated with LPS for varying durations (0 min, 10 min, 30 min, 60 min), and groups first treated with LPS for these durations followed by the addition of acacetin (10 μM). For evaluating acacetin’s effect on NLRP3 inflammasome activation, the study encompassed a control group, an LPS treated group, an LPS + Nigericin group, and groups treated with LPS + Nigericin followed by different concentrations of acacetin (2.5 μM, 5 μM, and 10 μM). Further details are available in the figure legends.

### 2.2 Cell samples collection and proteins extraction

After the above treatments, supernatants from each group were collected and combined with an equal volume of methanol and a quarter volume of chloroform. This mixture was then vortexed thoroughly and centrifuged at 14,000 × g at room temperature for 5 min. Following centrifugation, the resulting tri-layered solution had proteins localized in the intermediate layer. The top layer was carefully discarded, and 500 μL of methanol was added to the remaining solution. This was followed by another centrifugation at 16,000 × g for 5 min, after which the protein precipitate settled at the bottom. The supernatant was removed, and the resultant pellet was air-dried for about 5 min to get rid of residual methanol. The dried protein pellet was then reconstituted in sample buffer and subjected to heating at 101°C in a metal bath for 10 min. The prepared protein sample was deemed fit either for immediate Western Blotting assays or storage at −20°C.

For the cellular proteins, cells were enzymatically digested using 0.25% Trypsin-EDTA (25200–056, Gibco, United States) and gathered. These cells were then resuspended in 100 μL of radioimmunoprecipitation assay (RIPA) buffer (AR0105, Boster Bio, China), ensuring a homogenous mixture. The cell-lysis mixture was allowed to incubate at 4°C for 60 min. A centrifugation step at 14,000 × g for 15 min at 4°C allowed us to collect the supernatant, which was subsequently mixed with an appropriate volume of 5 × SDS-PAGE loading buffer infused with β-mercaptoethanol. The denaturation of proteins was achieved by placing this mixture in a 100°C-water bath for 5 min. After this, a final centrifugation at 14,000 × g for 5 min was done to obtain a clear supernatant. Lastly, the protein content was quantified using the standard BCA method (Easy II Protein Quantitative Kit, DQ111-01, TransGen Biotech, China), from which the volume equivalent to 30 μg of protein was calculated, setting the stage for downstream experimental endeavors.

### 2.3 Western blot

Electrophoresis conditions: the stacking gel was set at 80 V and the separating gel at 100 V. Following electrophoresis, proteins from the gel were transferred onto PVDF membranes (ISEQ00010/IPVH00010, Millipore, United States). Then the membrane was blocked with 5% bovine serum albumin (BSA) in TBST at room temperature for 2 h. It was then incubated with primary antibodies overnight at 4°C (Table 1). After washing thrice with TBST for 10 min each, the membrane was incubated with corresponding secondary antibodies (diluted 1:5,000) at room temperature for 2 h. Another three 10-min TBST washes were done, followed by an application of chemiluminescent reagent (mixed 1:1 of Solution A and B, 2 mL each) on the membrane. Protein bands were detected and captured using a chemiluminescence imaging system (Chemiscope 3000, Clinx, China). The relative expression level was calculated based on the absorbance ratio of the target gene band to the β-actin band.

**TABLE 1:** Primary antibodies used in Western blot analysis.

Protein	Cat no.	Dilution	Transfer time (min)	Company Info
NLRP3	ab263899	1:1000	120	abcam, United Kingdom
Caspase-1	ab179515	1:1000	60	abcam, United Kingdom
IL-1β	ab9722	1:500	60	abcam, United Kingdom
NF-κB p65	ab32536	1:800	60	abcam, United Kingdom
Phospho-NF-κB p65	ab76302	1:800	60	abcam, United Kingdom
IκBα	ab32518	1:1000	60	abcam, United Kingdom
Phospho-IκBα	5209s	1:800	60	CST, United States
ERK	ab184699	1:800	60	abcam, United Kingdom
Phospho-ERK	ab201015	1:800	60	abcam, United Kingdom
JNK	ab179461	1:1000	60	abcam, United Kingdom
Phospho-JNK	ab76572	1:800	60	abcam, United Kingdom
p38	ab170099	1:1000	60	abcam, United Kingdom
Phospho-p38	ab195049	1:800	60	abcam, United Kingdom
β-Actin	100166-MM10	1:1000	60	Sino Biological, China

### 2.4 Enzyme-linked immunosorbent assay

Cytokine levels of IL-1β, IL-18, and TNF-α in collected cell supernatants were measured using ELISA kits (EK201B/3-48, EK218-48 and EK282/3-48, LiankeBio, China) following the manufacturer’s instructions. After adding a stop solution to terminate the reaction, the color change was measured spectrophotometrically at a dual wavelength of 450 nm (maximum absorption wavelength) and 570 nm (reference wavelength) using a microplate reader (xMarkTM, Bio-Rad, United States). The optical density (OD) at 570 nm was subtracted from the OD at 450 nm to correct for optical imperfections. The corrected OD values were used to determine the concentrations of the cytokines in the supernatants by comparing them to the standard curve.

### 2.5 LDH release assay

To quantify the level of cellular cytotoxicity and necrosis, a LDH release assay was performed following the manufacturer’s instructions. LDH is a stable enzyme, released upon cell lysis, and it serves as a marker of cellular damage or death. Supernatants from each group were collected and assayed for LDH activity. The LDH assay was conducted using the LDH Cytotoxicity Detection Kit (A020-2, NJJCBio, China). This kit utilizes the conversion of lactate to pyruvate by LDH, with simultaneous conversion of a tetrazolium salt (INT) into a red formazan product. The amount of formazan product, which is directly proportional to the LDH activity in the sample, can be quantified by measuring the absorbance at 450 nm using a microplate reader. The calculation formula of LDH activity is:

$$\text{LDH Activity }\left(U/L\right)=\frac{\text{Absorbance}\;\text{of}\;\text{sample}\;-\;\text{Absorbance}\;\text{of}\;\text{blank}}{\text{Absorbance}\;\text{of}\;\text{standard}\;-\;\text{Absorbance}\;\text{of}\;\text{standard}\;\text{blank}}\times \text{Standard concentration}\times 1000$$

### 2.6 NF-κB nuclear translocation

The nuclear translocation of NF-κB was visualized using laser scanning confocal microscopy (LSCM). After the above treatments, the coverslips were thoroughly washed with three 2-min wash cycles using phosphate-buffered saline (PBS). Subsequent steps were fixation with 4% paraformaldehyde for 20 min, permeabilization with 0.5% Triton X-100 for 5 min, and blocking with 1% BSA for 30 min, each followed by identical wash cycles. Following blocking, cells were incubated at 37°C with primary NF-κB p65 antibody (2 μg/mL, ab76302, Abcam, United Kingdom) for 2 h. A post-incubation wash cycle was performed before the cells were exposed to a goat anti-rabbit IgG H&L secondary antibody (2 μg/mL, ab205718, United Kingdom) for 1 h at 37°C in the dark. Another wash cycle preceded DAPI counterstaining (1 μg/mL, C0065, Solarbio, Beijing, China) for 5 min at room temperature. After a final wash cycle, cells were mounted with 50% glycerol and imaged using a laser scanning confocal microscope (LSM700, Carl Zeiss AG, Germany).

### 2.7 K + ions concentration

Prepared cell suspensions were centrifuged at 110 *g* for 10 min, discarding the supernatant and retaining the cellular pellet. Cells were then washed twice with PBS, followed by centrifugation at 110 *g* for 10 min, retaining the cellular pellet. The cellular pellet was resuspended in 0.2 mL of deionized water and homogenized in an ice bath. Homogenates were then centrifuged at 450 *g* for 5 min, and 20 μL of the supernatant was mixed with 180 μL of protein precipitant. This was further centrifuged at 1370 *g* for 5 min, and 50 μL of the supernatant was collected for potassium measurement. Protein concentration in the samples was also determined to normalize the K+ ions concentration. The concentration of K+ ions was measured using a potassium ion assay kit (C001-2-1, NJJCBio, China), following the manufacturer’s instructions. The calculation formula of the K+ ions concentration in mmol/g of protein (mmol/gprot) is:

$$\begin{array}{ll} \text{Potassium ions concentration }\left(\text{mmol}/\text{gprot}\right) & =\frac{\text{Absorbance}\;\text{of}\;\text{sample}\;–\;\text{Absorbance}\;\text{of}\;\text{blank}}{\text{Absorbance}\;\text{of}\;\text{standard}\;–\;\text{Absorbance}\;\text{of}\;\text{blank}}\times \text{standard concentration}\\ & \times \text{sample dilution factor}÷\text{protein concentration in sample} \end{array}$$

### 2.8 Cl- ions concentration

The concentration of Cl-ions was determined using MQAE fluorescent indicator. After the previous preparation, the supernatant was discarded. The cells were then lysed with ultrapure water and incubated at 37°C for 15 min. The lysate was transferred to a new 1.5 mL EP tube and placed at −80°C for 30 min. After centrifuging at 6200 × g for 5 min, the supernatant was transferred to a fresh 1.5 mL EP tube. An equal volume of 50 μL of MQAE (HY-D0090, MedChemExpress, United States) at a concentration of 10 μM was added to the supernatant, and the mixture was vortexed to ensure uniform mixing. Then, 80 μL of the mixed solution was dispensed into a 96-well plate. Fluorescence intensity was measured using a fluorometer (VLB000D2, ThermoFisher, United States), with the excitation wavelength set at 355 nm and the emission wavelength at 460 nm.

### 2.9 Mitochondrial reactive oxygen species levels

The levels of mitochondrial ROS were evaluated using LSCM. Cells were adjusted to a concentration of 1 × 10^5^ cells/mL and plated onto adhesive glass coverslips (200 μL per coverslip). Following the intervention, cells were incubated with 100 μL MitoSOX Red (M36008, ThermoFisher, United States) mitochondrial superoxide indicator at a final concentration of 5 μM for 5 min at 37°C. Then, cells were washed with a three 2-min wash cycle using PBS. Subsequently, cells were fixed at room temperature for 20 min using 4% paraformaldehyde, followed by another wash cycle. The nuclei were counterstained with DAPI (1 μg/mL) in a dark room for 5 min at room temperature, followed by a final wash cycle. Coverslips were finally mounted using 50% glycerol and cells were visualized using a laser scanning confocal microscope. Quantification of ROS levels was performed using ImageJ software (National Institutes of Health, United States).

### 2.10 ASC oligomerization assay

Cells were collected and lysed in 100 µL of RIPA buffer (supplemented with protease and phosphatase inhibitors). The lysate was incubated in an ice bath for 60 min before centrifugation at 14000 *g*, 4°C for 15 min, with the supernatant collected for further processing. The cell lysate was then treated for oligomerized protein processing. The cells were digested with trypsin (25200–056, Gibco, United States) and resuspended in 0.5 mL of ice-cold buffer A. The lysate was centrifuged at 1800 *g* at 4°C for 8 min to remove the precipitate. A 30 μL aliquot of lysate was reserved for Western Blot of ASC as input controls. The remaining supernatant was diluted in a 1:1 ratio with buffer A and centrifuged at 2000 *g* at 4°C for 5 min. The supernatant was collected and diluted with an equal volume of CHAPS buffer before a centrifugation at 5000 *g* for 8 min to precipitate ASC oligomers. The supernatant was discarded and the pellet was resuspended in 50 µL of CHAPS buffer containing 4 mM of DSS (S1885, Sigma, United States) and incubated at room temperature for 30 min for protein cross-linking. The mixture was then centrifuged again at 5000 × g for 8 min at 4°C. The supernatant was discarded, and the pellet was resuspended in 30 µL of 2× protein loading buffer and denatured at 90°C for 2 min. Subsequent steps were conducted according to standard Western Blot procedures with anti-ASC antibody (sc-514414, Santa Cruz, United States).

### 2.11 Statistical analysis

Data were analyzed using GraphPad Prism 9 software (GraphPad Software Inc., United States). The Shapiro-Wilk test was used for normality, while the Brown-Forsythe test was applied to test for homogeneity of variances. For multiple comparisons, one-way analysis of variance (ANOVA) was used, and *post hoc* pairwise comparisons were conducted using the Least Significant Difference (LSD) method. Data are presented as means ± standard error of the mean (SEM). A *p*-value of less than 0.05 was considered statistically significant.

## 3 Results

### 3.1 Effect of acacetin on NLRP3 inflammasome activation induced by Nigericin

**FIGURE 1:**
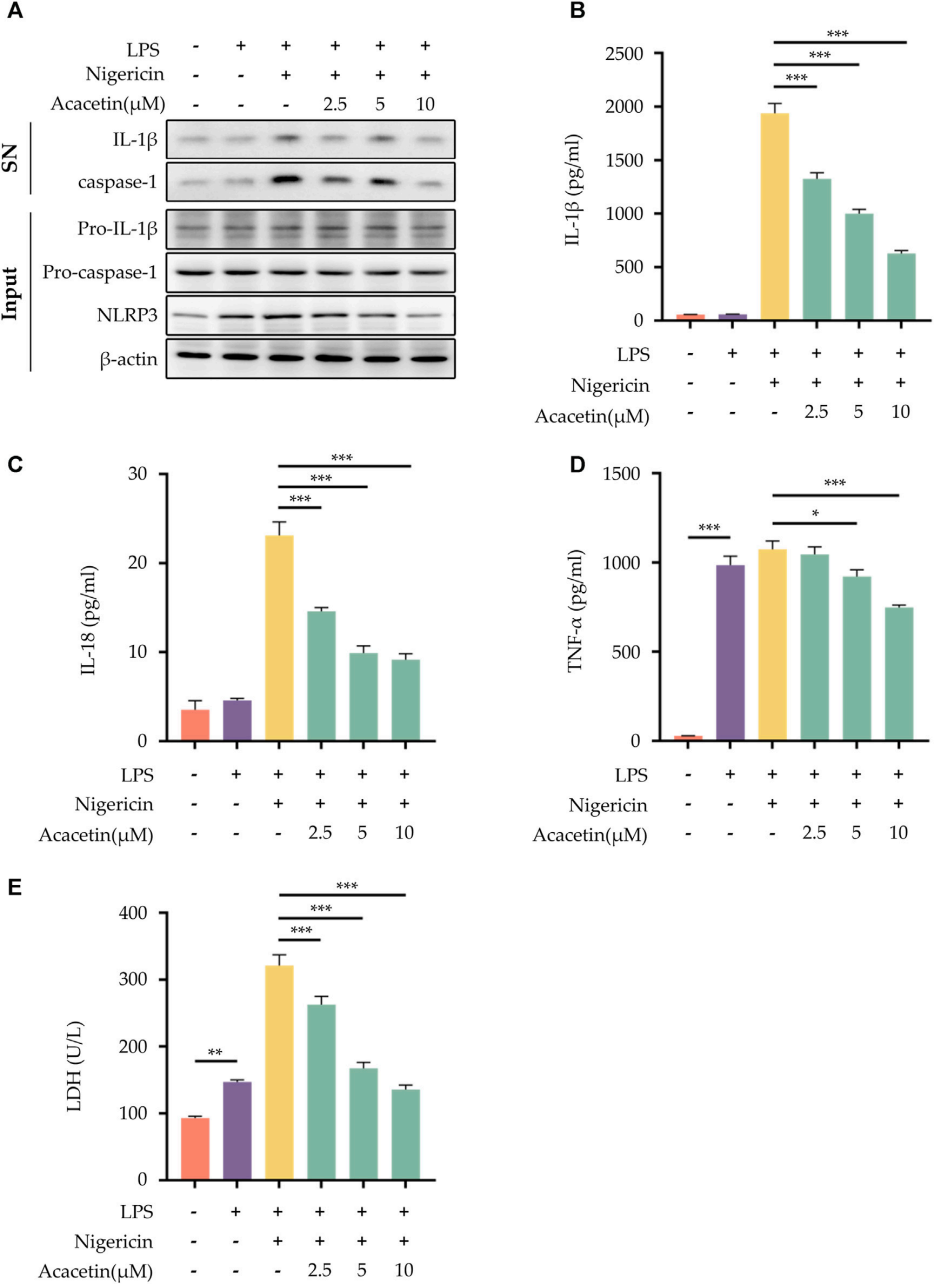
Effect of Acacetin on NLRP3 inflammasome activation induced by Nigericin. After pretreatment with LPS (50 ng/mL) for 3 h, BMDMs were treated with different concentrations of Acacetin (2.5 μM, 5 μM, and 10 μM) for 0.5 h, and then Nigericin (10 μM) was added for stimulation for 0.5 h. The culture supernatant and cell lysate were collected. **(A)** Western blot detected IL-1β and caspase-1 in the supernatant, and pro-IL-1β, pro-caspase-1, and NLRP3 in the cell lysate. **(B–D)** ELISA detected levels of IL-1β **(B)**, IL-18 **(C)**, and TNF-α **(D)** in the supernatant. **(E)** LDH activity was assessed. The data is presented as mean ± SEM (*n* = 3) and was analyzed with ANOVA followed by LSD. *p < 0.05, **p < 0.01, ***p < 0.001.

To investigate whether Acacetin could inhibit the activation of the NLRP3 inflammasome, several methods were conducted. Western blot analysis was used to quantify the expression levels of pro-caspase-1, pro-IL-1β, and NLRP3 in the cell lysates, as well as caspase-1 and IL-1β in the cell supernatant (Figure 1A). ELISA was utilized to measure the expression of TNF-α, IL-1β, and IL-18 in the supernatant (Figures 1B–D), and the LDH assay was conducted to evaluate cell death (Figure 1E). The results revealed that, compared to the control group, Nigericin induced the activation of NLRP3 inflammasome, leading to a significant increase in the expression of caspase-1, IL-1β, and NLRP3 in the supernatant and cell lysates. Intervention with Acacetin effectively reduced the expression of caspase-1, IL-1β, and NLRP3. In comparison to the control group, the expression of inflammatory cytokines TNF-α, IL-1β, IL-18, and LDH release was significantly elevated, while acacetin inhibited the expression of them. It indicates acacetin’s inhibition of Nigericin-induced NLRP3 inflammation activation. Among the tested concentrations, the inhibitory effect was most prominent at a dose of 10 μM. Consequently, this dose was selected for subsequent studies.

### 3.2 Effect of acacetin on NLRP3 inflammasome activation induced by multiple agonists

**FIGURE 2:**
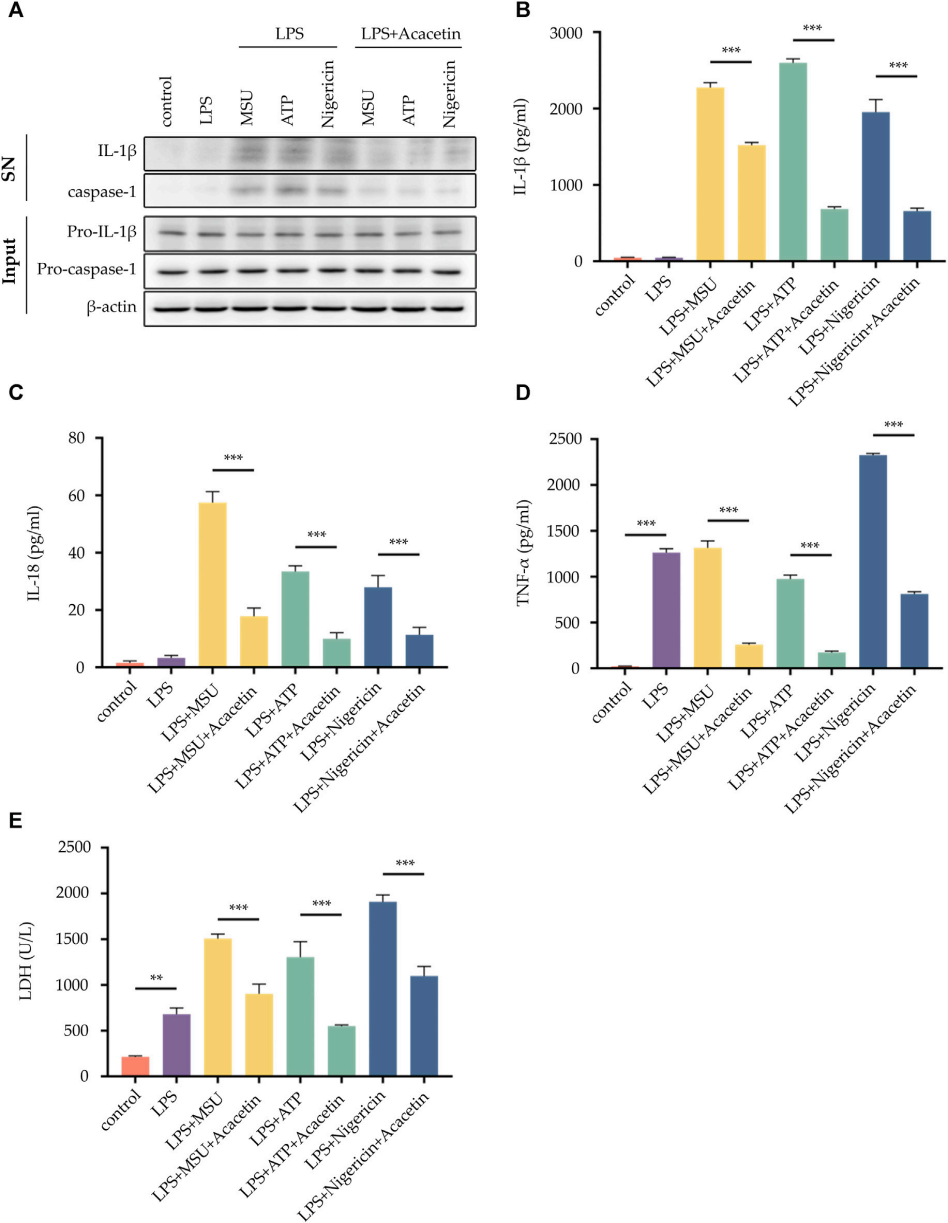
Effect of Acacetin on NLRP3 inflammasome activation induced by multiple agonists. BMDMs cells were pretreated with LPS (50 ng/mL) for 3 h and then treated with Acacetin (10 μM) for 0.5 h. After that, the cells were stimulated with MSU (150 μg/mL for 4 h), ATP (2.5 mM for 0.5 h), and Nigericin (10 μM for 0.5 h). The culture supernatant and cell lysate were collected. **(A)** Western blot detected IL-1β and caspase-1 in the supernatant, and pro-IL-1β and pro-caspase-1 in the cell lysate. **(B–D)** ELISA detected the levels of IL-1β **(B)**, IL-18 **(C)** and TNF-α **(D)** in the supernatant. **(E)** LDH activity was assessed. The data is presented as mean ± SEM (*n* = 3) and was analyzed with ANOVA followed by LSD *p < 0.05, **p < 0.01, ***p < 0.001.

We further investigated the effects of Acacetin on NLRP3 inflammasome activation induced by the canonical activators MSU, ATP, and Nigericin. The results revealed that compared to the control group, all three activators—MSU, ATP, and Nigericin—were able to induce the activation of NLRP3 inflammasome, leading to an increase in the expression of caspase-1 and IL-1β (Figure 2A). There was also an elevation in the release of TNF-α, IL-1β, IL-18, and LDH activity (Figures 2B–E). Remarkably, acacetin demonstrated an inhibitory effect on NLRP3 inflammasome activation by these canonical activators and decreased the secretion of TNF-α, IL-1β, IL-18, and LDH release.

### 3.3 Effect of acacetin on non-canonical NLRP3 inflammasome activation

**FIGURE 3:**
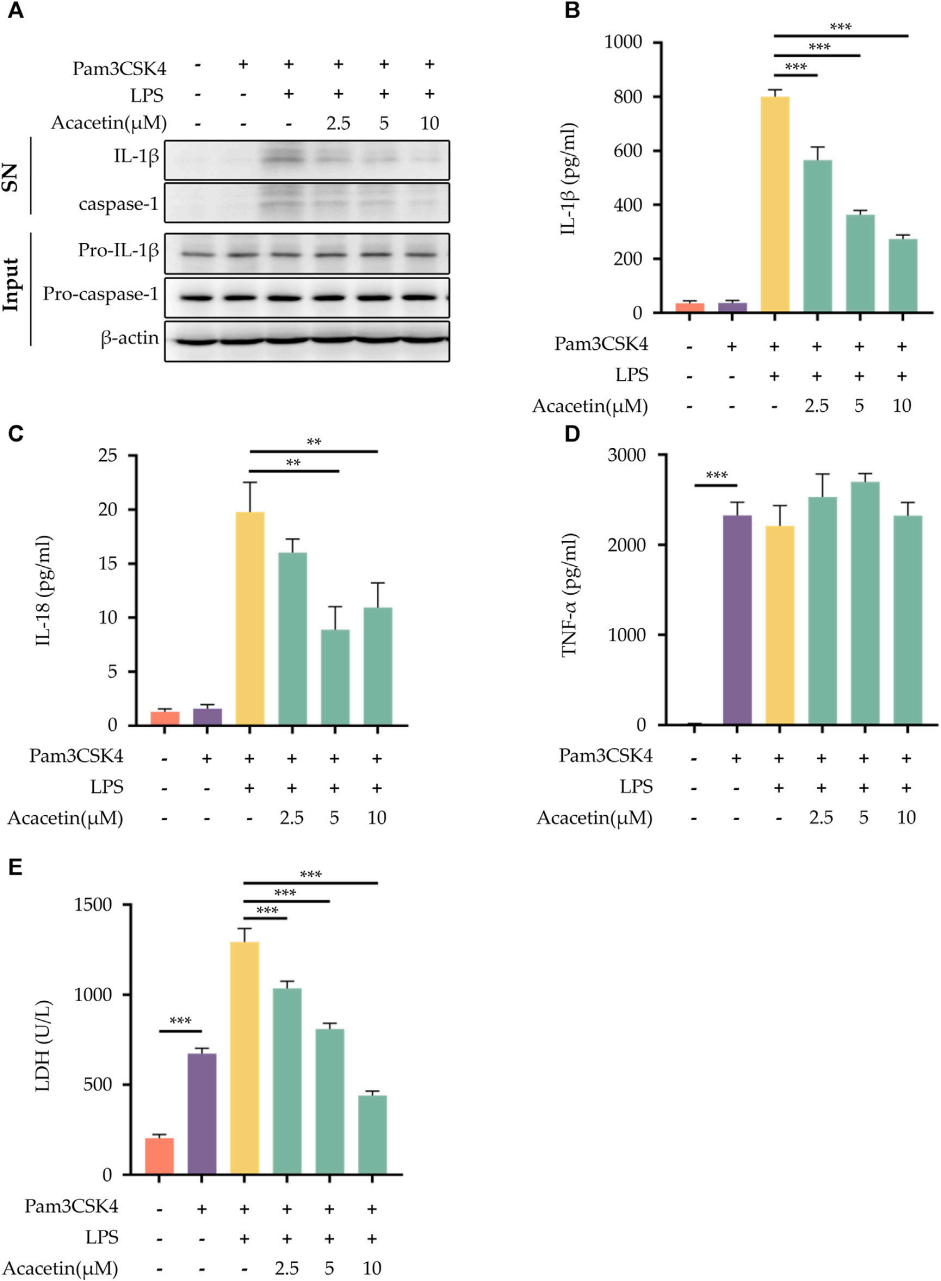
Effect of Acacetin on non-canonical NLRP3 inflammasome activation. After pretreatment with Pam3CSK4 (400 ng/mL) for 3 h, BMDMs were treated with acacetin of different concentrations (2.5 μM, 5 μM, and 10 μM) for 0.5 h. Subsequently, the cells were transfected with LPS (500 ng/mL) using Lipofectamine 3000 for 16 h. The culture supernatant and cell lysate were collected. **(A)** Western blot detected IL-1β and caspase-1 in the supernatant, and pro-IL-1β and pro-caspase-1 in the cell lysate. **(B–D)** ELISA detected the levels of IL-1β **(B)**, IL-18 **(C)** and TNF-α **(D)** in the supernatant. **(E)** LDH activity was also assessed. The data is presented as mean ± SEM (*n* = 3) and was analyzed with ANOVA followed by LSD. *p < 0.05, **p < 0.01, ***p < 0.001.

To explore the potential effects of acacetin on non-canonical NLRP3 inflammasome activation, BMDMs were pre-treated with Pam3CSK4 and then stimulated with LPS. Our findings demonstrated that acacetin effectively interrupted the cleavage of caspase-1 during non-canonical NLRP3 inflammasome activation. This interruption led to a subsequent inhibition of the secretion of IL-1β and IL-18, along with the release of LDH. Interestingly, the expression of TNF-α remained unaffected by acacetin under these conditions. Figure 3 summarizes these results.

### 3.4 Effect of acacetin on the NF-κB pathway

**FIGURE 4:**
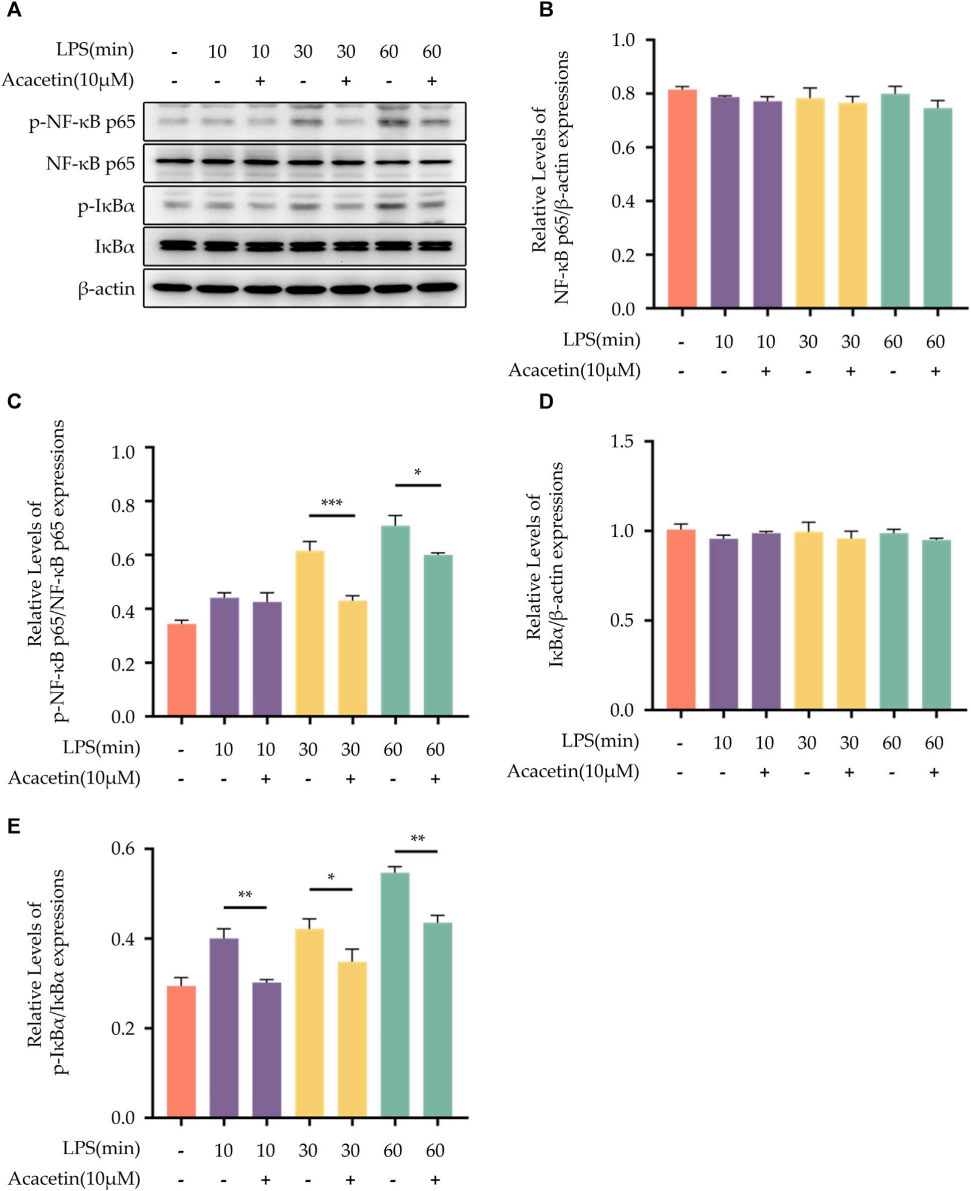
Effect of Acacetin on the NF-kB pathway. BMDMs were primed with LPS for different durations (0, 10 min, 30 min, and 60 min) and then treated with acacetin (10 μM). **(A)** Western blot was used to detect the relative expression levels of NF-κB p65 **(B)**, p- NF-κB p65 **(C)**, IκBα **(D)**, and p-IκBα **(E)** in the cells. The data is presented as mean ± SEM (n = 3) and was analyzed with ANOVA followed by LSD. *p < 0.05, **p < 0.01, ***p < 0.001.

Western Blot was employed to detect the protein expression levels of phosphorylated p65 (p-p65), p65, phosphorylated IκBα (p-IκBα), and IκBα. As shown in Figure 4, the protein expression levels of NF-κB p65 and IκBα did not differ in all groups. Compared to the control group, the expression levels of p-65 and p-IκBα were upregulated with the progression of LPS stimulation time. Upon treatment with acacetin, the expression of both p-65 and p-IκBα was markedly reduced.

### 3.5 Effect of acacetin on nuclear translocation of NF-κB p65

**FIGURE 5:**
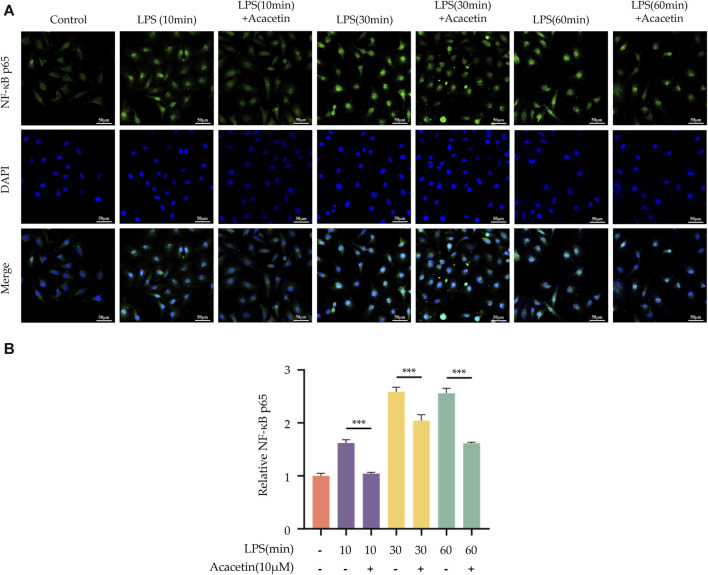
Effect of Acacetin on nuclear localization of NF-κB p65. BMDMs were primed with LPS for different durations (0, 10 min, 30 min, and 60 min) and then treated with acacetin (10 μM). **(A)** Immunofluorescence staining of NF-κB p65 was performed. NF-κB p65 is depicted in green, and the cell nuclei are shown in blue. Scale bar: 50 μm. **(B)** Quantification of relative fluorescence intensity of NF-κB p65. The data is presented as mean ± SEM (*n* = 3) and was analyzed with ANOVA followed by LSD. ***p < 0.001.

The results from LSCM revealed that in the blank control group, NF-κB p65 (green) was broadly distributed within the cytoplasm, with no observable localization within the cell nucleus (blue). Upon LPS stimulation, a substantial amount of NF-κB p65 was observed to localize to the cell nucleus, indicating that LPS promotes the activation and nuclear translocation of NF-κB p65. Following treatment with acacetin, the levels of LPS-mediated NF-κB p65 localized to the nucleus were significantly reduced, indicating that acacetin significantly inhibits LPS-mediated NF-κB p65 nuclear translocation. A visual overview of the results is given in Figure 5.

### 3.6 Effect of acacetin on the mitogen-activated protein kinase pathway

**FIGURE 6:**
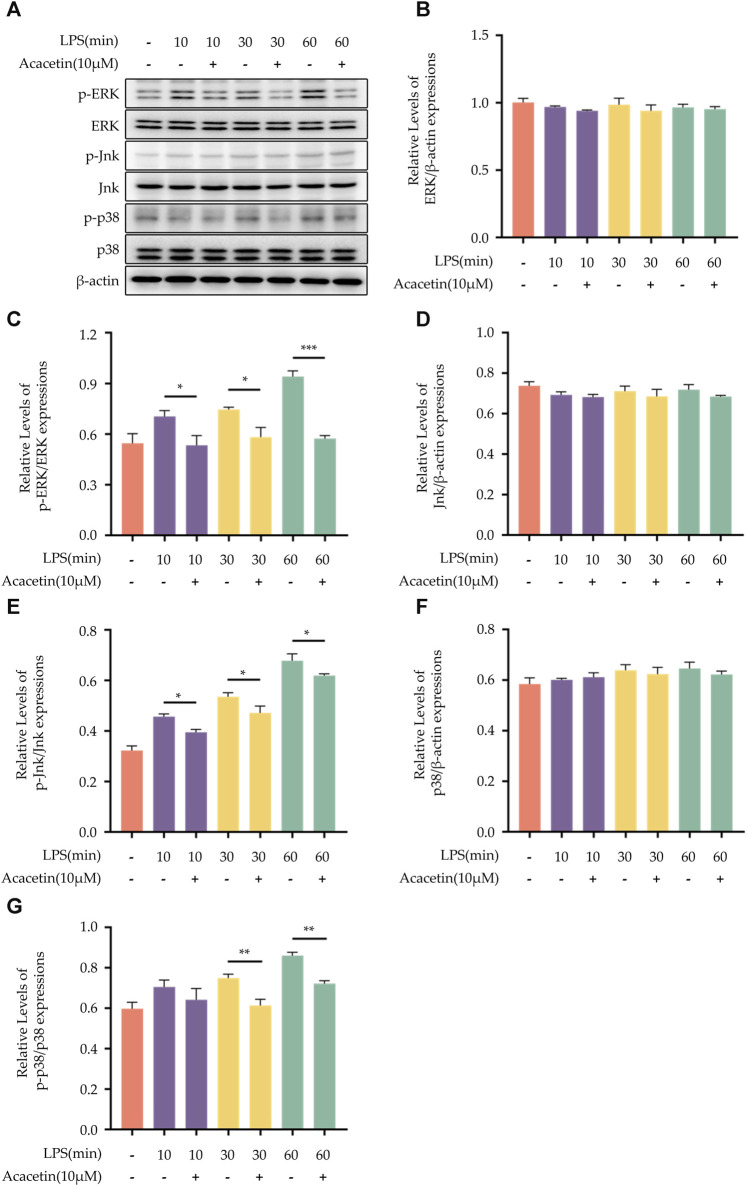
Effect of Acacetin on the MAPK pathway. BMDMs were primed with LPS for different durations (0, 10 min, 30 min, and 60 min), then treated with acacetin (10 μM). **(A)** Western blot was conducted to detect the relative expressions of ERK **(B)**, p-ERK **(C)**, JNK **(D)**, p-JNK **(E)**, p38 **(F)**, and p-p38 **(G)** in the cells. The data is presented as mean ± SEM (n = 3) and was analyzed with ANOVA followed by LSD. *p < 0.05, **p < 0.01, ***p < 0.001.

The MAPK family signaling pathways include p38, ERK, and JNK. To assess their activity, Western blot analysis was conducted to measure the expression levels of phosphorylated ERK (p-ERK), ERK, phosphorylated JNK (p-JNK), JNK, phosphorylated p38 (p-p38), and p38. In the blank control group, the expression levels of p-ERK, p-JNK, and p-p38 were found to be at a low level. Upon LPS stimulation, there was an increase in the expression of these phosphorylated proteins. Acacetin effectively inhibited the LPS-induced expression of p-ERK, p-JNK, and p-p38, underscoring its modulatory effect on the MAPK pathway. The results are shown in Figure 6.

### 3.7 Effect of acacetin on K+ and Cl- ions efflux during NLRP3 inflammasome activation

**FIGURE 7:**
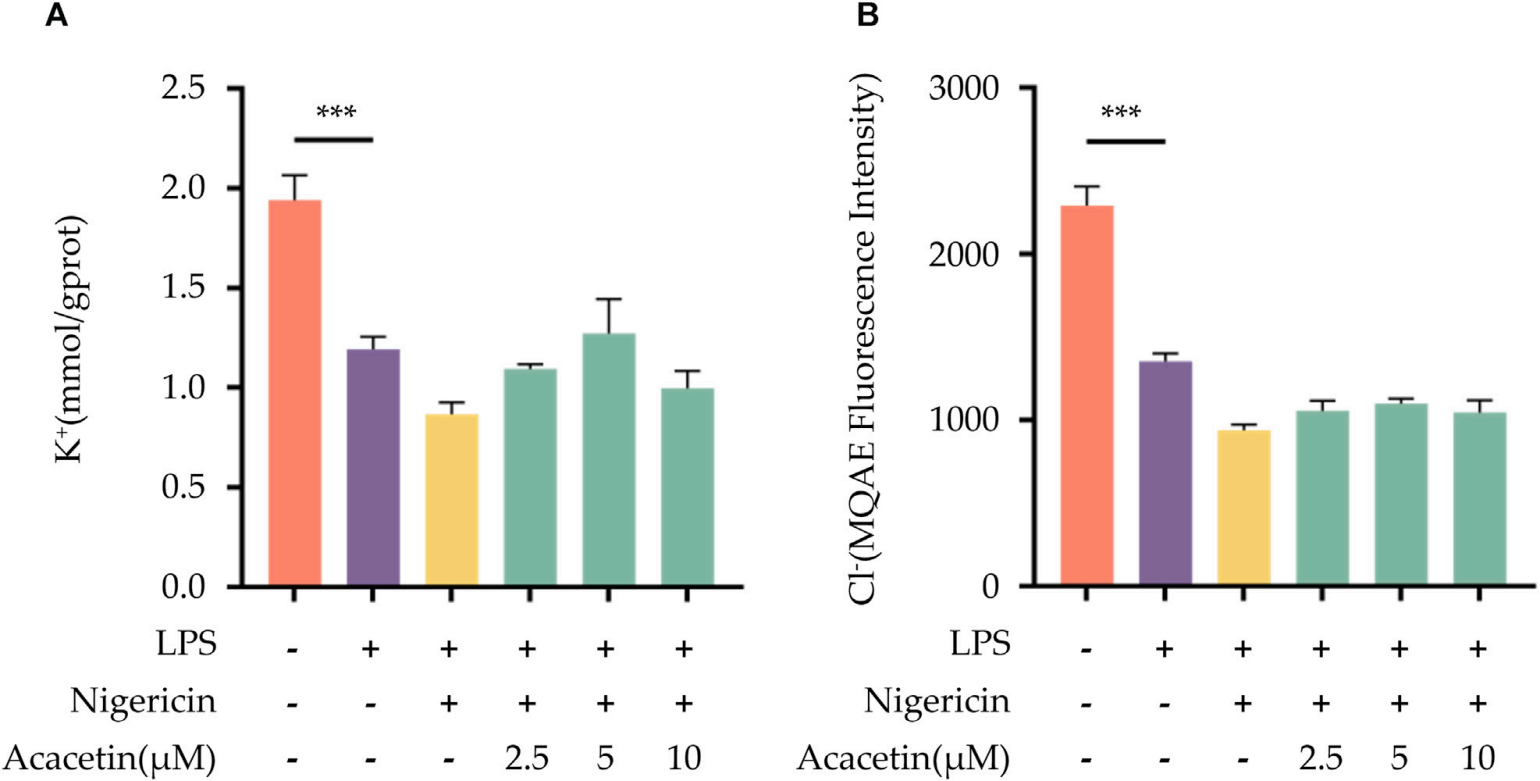
Effect of Acacetin on K+ and Cl- Ions Efflux During NLRP3 Inflammasome Activation. BMDMs were primed with LPS (50 ng/mL) for 3 h, followed by treatment with different concentrations of acacetin (2.5 μM, 5 μM, and 10 μM) for 0.5 h, and then stimulated with Nigericin (10 μM) for 0.5 h. The cell suspension was collected and processed. **(A)** K+ ions concentration was measured using a potassium ion assay kit (mmol/gprot). **(B)** Cl-ions concentration was determined using MQAE fluorescent indicator. The data is presented as mean ± SEM (*n* = 3) and was analyzed with ANOVA followed by LSD. *p < 0.05, **p < 0.01, ***p < 0.001.

Many studies have highlighted the important role of K+ and Cl-ions in the activation process of the NLRP3 inflammasome. In our investigation, compared to the blank control group, the expression of intracellular K+ and Cl-ions decreased in the LPS + Nigericin group. The intervention of acacetin did not reverse the reduction of K+ and Cl-ions within the cells, suggesting that acacetin does not affect the efflux of K+ and Cl-ions induced by Nigericin. Therefore, acacetin does not affect NLRP3 inflammasome activation through the efflux of K+ and Cl-ions. See Figure 7 for the results.

### 3.8 Effect of acacetin on mitochondrial damage and reactive oxygen species during NLRP3 inflammasome activation

**FIGURE 8:**
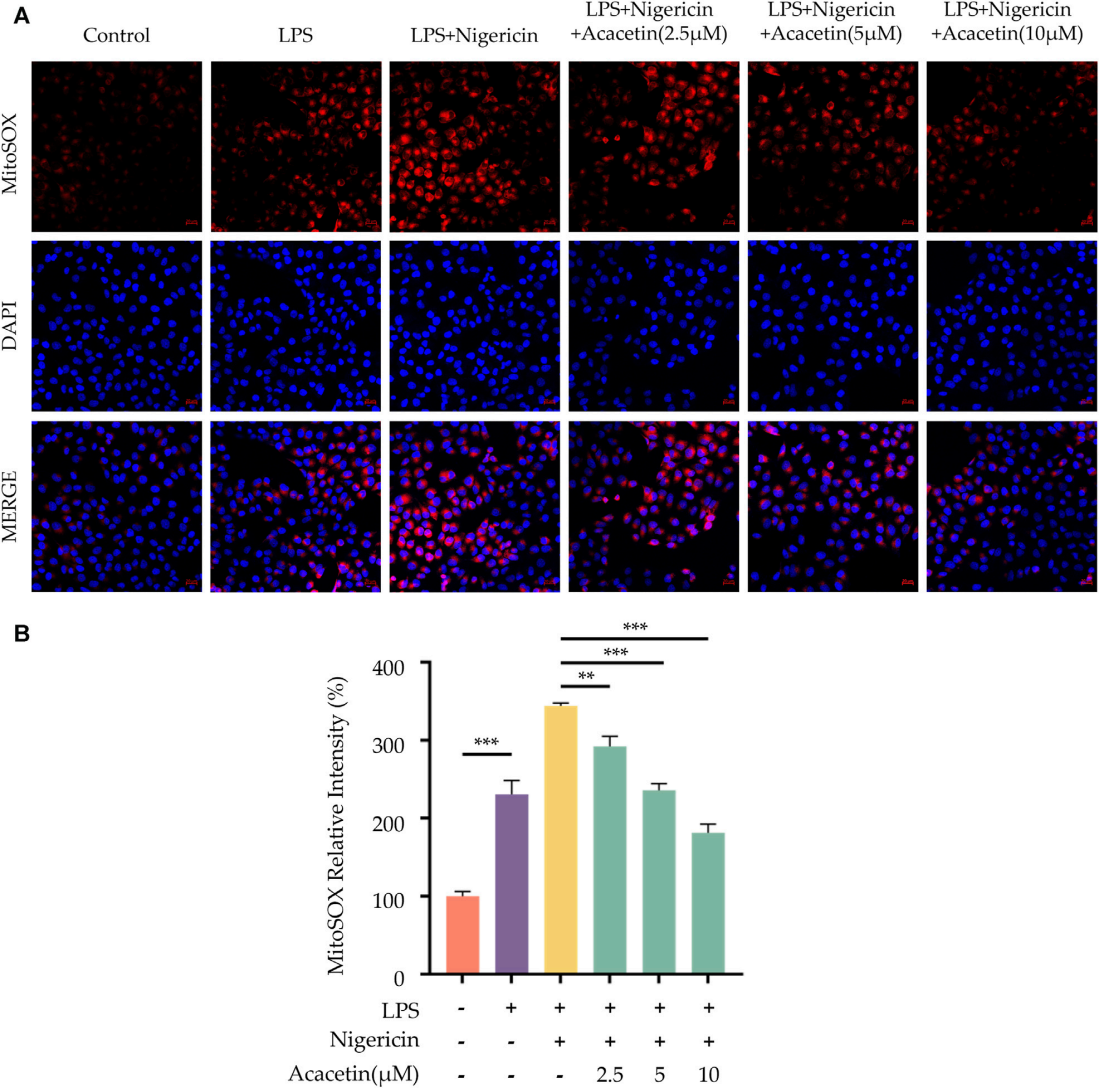
Effect of Acacetin on Mitochondrial Damage and ROS During NLRP3 Inflammasome Activation. BMDMs were primed with LPS (50 ng/mL) for 3 h, followed by treatment with different concentrations of acacetin (2.5 μM, 5 μM, and 10 μM) for 0.5 h, and then stimulated with Nigericin (10 μM) for 0.5 h. The cells were then incubated with MitoSOX Red Indicator (5 μM) to detect the levels of mitochondrial ROS. The images **(A)** were acquired using laser scanning confocal microscopy and the bar plot **(B)** was quantitated by fluorescence intensity. Scale bar: 20 μm. The data is presented as mean ± SEM (*n* = 3) and was analyzed with ANOVA followed by LSD. *p < 0.05, **p < 0.01, ***p < 0.001.

ROS are key upstream signals in the NLRP3 inflammasome activation. We utilized MitoSOX to label ROS and observed their expression levels in cells using a laser confocal microscope. The study found compared to the control group, ROS production increased following Nigericin induction. Intervention with acacetin subsequently led to reduced ROS production in a dose-dependent manner. Thus, acacetin may influence NLRP3 inflammasome activation by modulating ROS production (Figure 8).

### 3.9 Effect of acacetin on ASC aggregation during NLRP3 inflammasome activation

**FIGURE 9:**
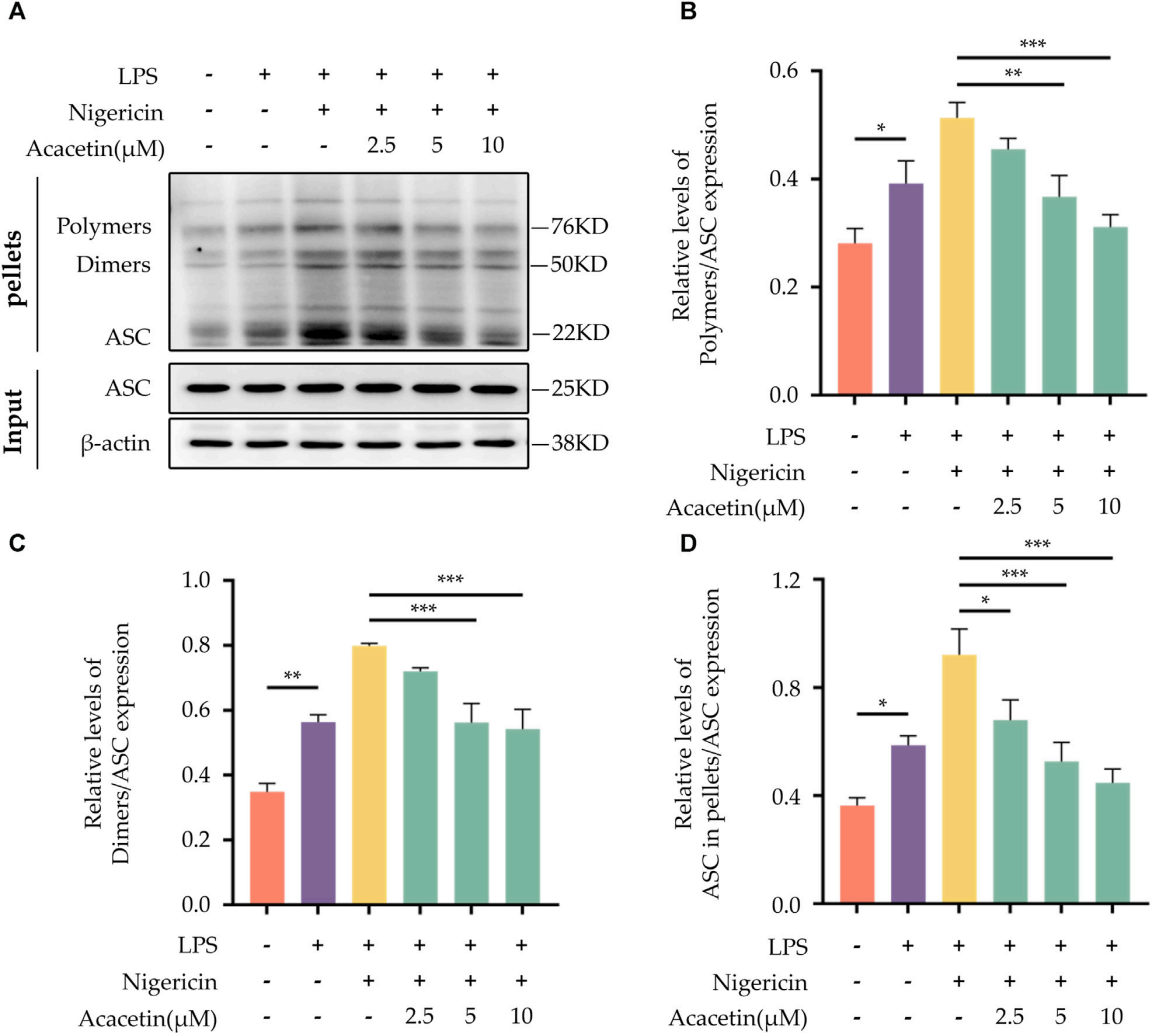
Effect of Acacetin on ASC Aggregation During NLRP3 Inflammasome Activation. BMDMs were primed with LPS (50 ng/mL) for 3 h, followed by treatment with different concentrations of acacetin (2.5 μM, 5 μM, and 10 μM) for 0.5 h, and then stimulated with Nigericin (10 μM) for 0.5 h. The cells were lysed with pellets crosslinked with DSS to detect ASC aggregation. **(A)** Western blot was performed to detect the relative expression of Polymers **(B)**, Dimers **(C)**, and ASC in pellets **(D)** in the cells, with ASC in INPUT serving as the loading control. The data is presented as mean ± SEM (n = 3) and was analyzed with ANOVA followed by LSD. *p < 0.05, **p < 0.01, ***p < 0.001.

ASC aggregation is an important hallmark of NLRP3 inflammasome activation. In comparison with the control group, Nigericin was observed to induce ASC oligomerization. Treatment with Acacetin led to a dose-dependent reduction in ASC oligomerization. The outcome is illustrated in Figure 9.

## 4 Discussion

Acacetin has been recognized for its pharmacological properties, including antioxidant and anti-inflammatory effects, and has demonstrated therapeutic potential against various diseases in cellular or animal studies. Multiple studies have found that acacetin can exert protective effects on cardiovascular diseases by regulating pathways such as TGF-β/Smad3, MAPK, and PI3K/Akt, inhibiting the expression of inflammatory cytokines, and promoting the secretion of anti-inflammatory factors (Liu et al., 2016; Wu W.-Y. et al., 2018; Li Z.-Y. et al., 2023). Wu D. et al., 2018 found in cellular experiments that acacetin can inhibit the production of ROS, increase the activity of HO-1 and Nrf2, and suppress the expression of inflammatory factors TNF-α and IL-1β, thereby alleviating LPS-induced lung injury. *In vitro* studies by Ha et al. (Ha et al., 2012) showed that acacetin could suppress the expression of NO, iNOS, PGE2, COX2, TNF-α, and IL-1β in LPS-stimulated BV2 cells. Moreover, acacetin has been found to inhibit 6-OHDA-induced neuronal death and ROS-mediated apoptotic cascades, thereby exerting protective effects against Parkinson’s Disease (PD) (Kim et al., 2017). Wang et al. (Wang et al., 2015) discovered that acacetin could mediate the transcriptional regulation of APP and BACE-1, downregulate their protein expression, reduce Aβ formation, and also inhibit APP synthesis, thus lessening the formation of senile plaques and providing protection against Alzheimer’s Disease (AD). Although these studies confirm the anti-inflammatory effect of acacetin, the specific mechanisms remain to be further elucidated, and it is still unclear whether acacetin could become a candidate drug for inhibiting the NLRP3 inflammasome. In this study, we found that acacetin inhibits the activation of the NLRP3 inflammasome induced by canonical activators such as MSU, ATP, and Nigericin. Acacetin is able to suppress caspase-1 cleavage, inhibit the secretion of IL-1β and IL-18, reduce LDH activity, and furthermore, it can also inhibit non-classical NLRP3 inflammasome activation.

The activation of the NLRP3 inflammasome involves two steps—priming step and activation step. The priming step mainly activates the NF-κB pathway, thereby upregulating the expression of NLRP3 and Pro-IL-1β. The activation step primarily triggers the assembly of the NLRP3 inflammasome, leading to the secretion of IL-1β and IL-18 mediated by caspase-1, as well as pyroptosis (Bauernfeind et al., 2009; Swanson et al., 2019). LPS, a major outer membrane component of Gram-negative bacteria, binds to toll-like receptor 4 (TLR4), activates the MAPK pathway, and induces an NF-κB-dependent inflammatory cascade, resulting in the overproduction of pro-inflammatory mediators such as nitric oxide (NO) and prostaglandin E2 (PGE2) as well as cytokines like TNF-α and IL-6 (Zeng et al., 2017).

Here, we explored the role of acacetin during the NLRP3 inflammasome priming step by isolating and inducing mouse BMDMs with LPS. We found that LPS increased TNF-α secretion and NLRP3 expression in BMDMs. While acacetin could inhibit TNF-α secretion and reduce NLRP3 expression, indicating its influence on the priming of NLRP3 inflammasome activation. Several NLRP3 inflammasome inhibitors can regulate the priming process. Compounds such as curcumin (Hasanzadeh et al., 2020), resveratrol (Liu et al., 2019), green tea polyphenols (Wang et al., 2019), BAY11-7082 (Irrera et al., 2017), and INF-39 (Shi et al., 2021) have all shown inhibitory effects on the NF-κB pathway. Chloroquine (Chen et al., 2017), Sinigrin (Lee et al., 2017), and ECGG (Luo et al., 2021) can inhibit both NF-κB and MAPK pathways, thereby regulating the transcriptional levels of NLRP3 protein components and modulating NLRP3 inflammasome activation. Whether acacetin plays an inhibitory role during the priming is not yet clear, and whether it inhibits NLRP3 inflammasome activation by suppressing the NF-κB and MAPK pathways remains unreported. In our study, we discovered that acacetin can inhibit the expression of p-65 and p-IκBα, suppress nuclear translocation of NF-κB, and inhibit the expression of p-ERK, p-JNK, and p-P38, thus modulating the priming process.

Following the priming step, the activation step of the NLRP3 inflammasome is marked by pivotal roles of ASC aggregation, ROS production, K+ and Cl-ions efflux. Various inhibitors of NLRP3, such as MNS (He et al., 2014), NBC6 (Baldwin et al., 2017), and BOT-4-one (Shim et al., 2017), have been shown to prevent the formation of ASC specks during the activation of the NLRP3 inflammasome. Youm et al. (He et al., 2018) demonstrated that β-hydroxybutyrate (BHB) could specifically inhibit NLRP3 inflammasome activation by suppressing K+ ions efflux. Similarly, MCC950 was found to attenuate Cl-ions efflux during NLRP3 inflammasome activation (Jiang et al., 2017). Yang et al., 2016 pinpointed that sulforaphane can impede NLRP3 inflammasome activation via inhibiting unsaturated fatty acid-induced ROS production through the AMP-activated protein kinase (AMPK) autophagy pathway. In our previous studies (Bu et al., 2019; 2022; 2023), we identified that acacetin could diminish ROS level in microglial cells post-oxygen glucose deprivation (OGD) injury. However, whether acacetin affects ASC aggregation, as well as the efflux of K+ and Cl-ions, thereby modulating NLRP3 inflammasome activation, remains unreported. In this study, we observed that acacetin inhibited ASC aggregation and ROS production following NLRP3 inflammasome activation but did not alter the efflux of K+ and Cl-ions during the activation step.

In summary, this study has unveiled that acacetin can inhibit the activation of the NLRP3 inflammasome. Specifically, acacetin modulates both the priming and assembly of NLRP3 inflammasome activation by suppressing the NF-κB and MAPK pathways, subsequently hindering ASC aggregation and ROS production. While acacetin displays inhibitory effects on NLRP3 inflammasome activation, further studies are warranted to determine its protective role against diseases mediated by the NLRP3 inflammasome.

## Data Availability

The original contributions presented in the study are included in the article/Supplementary material, further inquiries can be directed to the corresponding authors.
